# Antibiotic prescriptions for outpatient acute rhinosinusitis in Canada, 2007-2013

**DOI:** 10.1371/journal.pone.0181957

**Published:** 2017-07-27

**Authors:** Prateek Sharma, Rita Finley, Scott Weese, Shiona Glass-Kaastra, Warren McIsaac

**Affiliations:** 1 Department of Pathobiology, Ontario Veterinary College, University of Guelph, Guelph, Ontario, Canada; 2 Centre for Food-borne, Environmental and Zoonotic Infectious Diseases, Public Health Agency of Canada, Guelph, Ontario, Canada; 3 Granovsky-Gluskin Family Medicine Centre, Ray D. Wolfe Department of Family Medicine, Mount Sinai Hospital, Toronto, Ontario, Canada; 4 Department of Community and Family Medicine, University of Toronto, Toronto, Ontario, Canada; California Northstate University College of Medicine, UNITED STATES

## Abstract

**Introduction:**

Acute rhinosinusitis (ARS) is a respiratory disease commonly caused by viral infections. Physicians regularly prescribe antibiotics despite bacterial etiologies being uncommon. This is of concern, as this use adds to the selection pressure for resistance. Here we present the descriptive epidemiology of acute rhinosinusitis and corresponding antibiotic prescribing practices by Canadian outpatient physicians from 2007–2013.

**Materials/Methods:**

Diagnosis and antibiotic prescription data for ARS were extracted from the Canadian Disease and Therapeutic Index for 2007 to 2013, and population data were acquired from Statistics Canada. ARS diagnosis and antibiotic prescription rates and frequencies of antibiotic classes were calculated.

**Results:**

Eighty-eight percent of patients diagnosed with ARS in 2013 were adults, with a greater rate of antibiotic prescriptions observed among the adults relative to the pediatric patients (1632.9 and 468.6 antibiotic prescriptions per 10,000 inhabitants). Between 2007 and 2013, the ARS diagnosis rate decreased from 596 to 464 diagnoses per 10,000 inhabitants, while the percentage of diagnoses with antibiotic prescriptions at the national level remained stable (87% to 84%). From 2007 to 2013, prescription rates for macrolides decreased from 203.5 to 105.4 prescriptions per 10,000 inhabitants. In 2013, penicillins with extended spectrum were more commonly prescribed compared to macrolides among adult patients (153.5 and 105.4 prescriptions per 10,000 inhabitants, respectively).

**Conclusion:**

This study is the first to describe physician antibiotic prescribing practices for treatment of ARS in Canada. Results show that antibiotic treatment for ARS represents an area for implementing antimicrobial stewardship, and through it, managing antibiotic resistance. Further work is required to better understand diagnosing practices and treatment criteria for ARS, and use this information to further assist physicians to limit unnecessary antibiotic prescribing practices.

## Introduction

There is global consensus that to address the problem of antibiotic resistance prudent management of antibiotics across all sectors is required. Since 2013, Canadian hospitals seeking accreditation have begun to develop antimicrobial stewardship programs, where antimicrobial therapy is monitored and regulated by a stewardship team [1]. This facilitates education about optimal prescribing practices and can help improve antimicrobial use practices [2,3]. In community settings, primary healthcare providers often do not have the benefit of such programs, and therefore receive little feedback regarding the appropriateness of their antibiotic prescriptions. As outpatient antibiotic use accounts for the majority of antibiotic prescriptions in Canada and diseases of the respiratory system were responsible for 46% of Canadian antibiotic recommendations in 2013, they represent an important area for antimicrobial stewardship research [4].

Acute rhinosinusitis (ARS) is a common respiratory condition seen by primary care health providers, with an incidence ranging from 15 to 40 episodes per 1000 patients per year [5]. It is typically characterized by purulent nasal drainage accompanied by nasal obstruction, facial pain-pressure-fullness, or both [5]. ARS is characterized by purulent nasal drainage accompanied by nasal obstruction, facial pain-pressure-fullness, or both [5]. Causes of ARS can be environmental from exposure to allergens or irritants, as well as a result from exposure to bacterial, fungal or viral pathogens [6]. However, viral agents account for 90–98% of ARS episodes [6]. Up to 2% of acute viral rhinosinusitis (AVRS) episodes are reported to be complicated by a secondary bacterial infection (superinfection), termed acute bacterial rhinosinusitis (ABRS) [7]. Persistent or severe rhinosinusitis symptoms or worsening after signs of improvement are clinical criteria of ABRS that distinguish it from AVRS in the Canadian Society of Otolaryngology—Head and Neck Surgery and the Infectious Disease Society of America consensus-derived treatment guidelines [6,7]. Furthermore, > 10^4^ colony forming units per mL from bacterial cultures of sinus aspirates is a laboratory criteria for ARS; however, sinus aspirates are an invasive procedure and not routinely recommended in primary care [7,8]. Antibiotics are used in ABRS treatment to eliminate the infection and prevent further complications, in addition to concomitant symptomatic management and are therefore warranted [6,7]. However, antibiotic treatment is not warranted in AVRS cases. Given that viral etiologies account for the majority of cases of ARS, prescribing practices by Canadian physicians should reflect the low occurrence of bacterial disease and recommendations from guidelines.

Studies from the United States indicate that there is a high utilization of antibiotics for treating sinus infections with over 80% of ARS patients having been prescribed an antibiotic, despite increasing evidence of their limited benefits [9–11]. Presently, there have not been any studies that evaluate how ARS is treated in the Canadian outpatient setting. The objectives of this study were to describe the demography and diagnosis-rate of outpatient acute rhinosinusitis and the corresponding antibiotic prescriptions by Canadian physicians treating ARS patients from 2007 to 2013.

## Materials and methods

The Canadian Disease and Therapeutic Index (CDTI) dataset (IMS Health Canada, Inc.) provides information about the patterns and treatments of disease encountered by office-based physicians across the country. Office-based physicians include general practice, internal medicine, obstetrics/gynecology, otolaryngology, ophthalmology, psychiatry, neurology, surgery, pediatrics and dermatology. For four consecutive quarters, 652 office-based physicians maintain a practice diary describing information on every patient visit during a randomly selected 48-hour period within that quarter. The participating physicians are provided a stipend for their participation and between 85% to 91% of them participate across all quarters [12]. A two-stage stratified sampling design is utilized. In the first stage, stratification is done at the province/region level and then secondarily by practice specialty. Projection factors (Horvitz-Thompson estimator) are used to create estimates that are representative of the approximately 55,000 office-based Canadian physicians [4]. In 2012, there were 47 physicians in the Eastern region (7.2%), 410 physicians in the Central region (62.9%), and 195 in the Western region (29.9%) that participated in the collection of this information. Overall, the dataset contains information regarding: patient age, gender, reason for visit, diagnosis, name(s) of the treatment(s) recommended or discussed (including drugs, referrals, environmental, behavioural, or dietary changes, etc.), desired therapeutic effect(s), and the presence of concomitant therapies.

IMS Health Canada, Inc. assigns International Classification of Diseases—Ninth Revision System (ICD-9) codes to the diagnostic information provided by physicians. In turn, the Public Health Agency of Canada further classifies the ICD-9 codes into disease-specific classes for further analysis [13]. For example, the disease category of “acute rhinosinusitis” extracted for the years 2007 to 2013 included the following ICD-9 codes: 461.0 Acute Maxillary Sinusitis; 461.2 Acute Ethmoidal Sinusitis; 461.8 Other Acute Sinusitis; and 461.9 Acute Sinusitis Unspecified. Antibiotic classes were classified using the Anatomical Therapeutic Chemical (ATC) classification system as identified by the World Health Organization [14].

The CDTI data were age stratified by IMS Health Canada, Inc. and aggregated for the following age-groups, 0–2 years, 3–9 years, 10–19 years, 20–39 years, 40–59 years, 60–64 years, and 65 years and older. For assessing regional differences, provinces were grouped into three geographical regions. The Eastern region included the provinces of New Brunswick, Newfoundland and Labrador, Nova Scotia, and Prince Edward Island. The Central region had the provinces of Ontario and Québec. British Columbia, Alberta, Saskatchewan and Manitoba represented the Western region.

Population size information was acquired from Statistics Canada consisting of the 2011 Canadian census estimates [15]. Both the ARS diagnosis and antibiotic prescribing rates were calculated. The ARS diagnosis rate was defined as the total number of ARS diagnoses divided by the population size during that time period. Similarly, the ARS prescription rate was defined as the total number of ARS prescriptions divided by the population size. It should be noted that drug prescriptions are not necessarily equivalent to antimicrobial use because the patient may not have left the physician’s office with a prescription or filled the prescription at the pharmacy.

Changes in the yearly percentage of patients recommended an antibiotic were used to estimate changes in physician behaviour over time. Due to the complexity of the data collection consisting of double-stratification and projection estimate methods, in addition to the small sample size of physicians included in the CDTI dataset, statistical comparisons between regions and over time are inappropriate and not possible; therefore, descriptive data alone is presented in this publication.

All data analysed in this study were anonymous. Data processing and visualization were performed using R [16].

## Results

**Fig 1:**
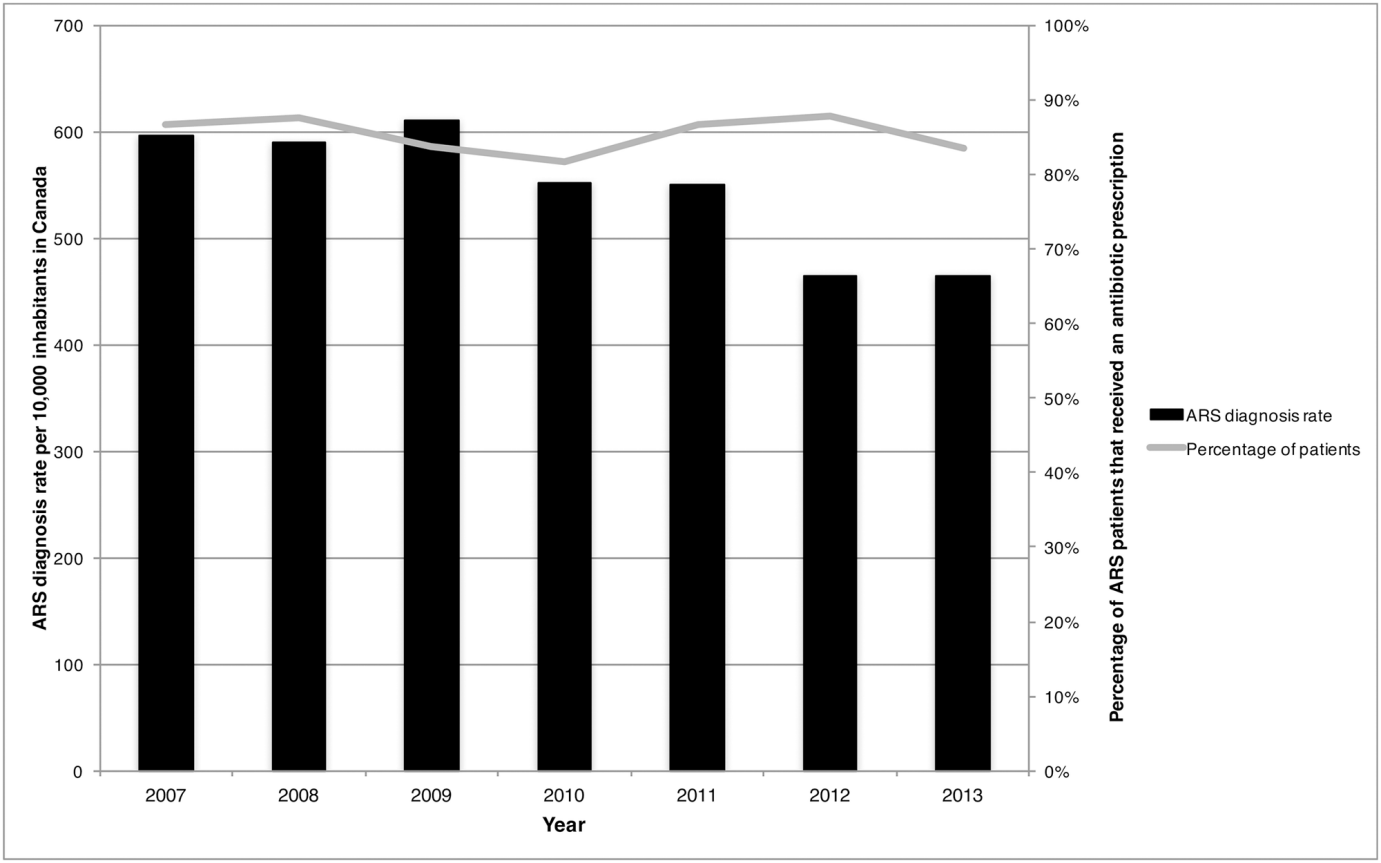
Acute rhinosinusitis diagnosis rate per 10,000 inhabitants and percentage of acute rhinosinusitis diagnoses with an antibiotic prescription by year in Canada, 2007–2013.

**Fig 2:**
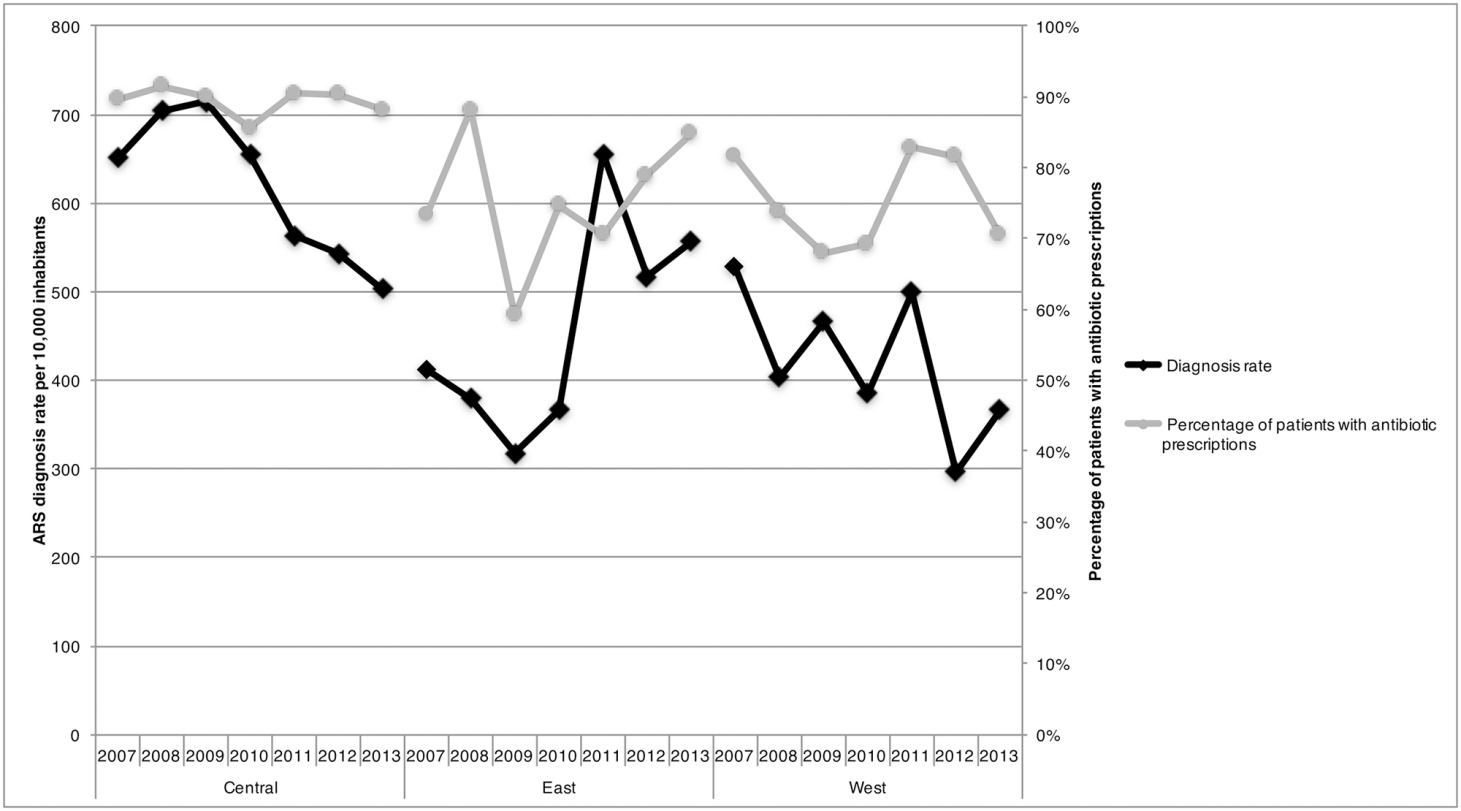
Acute rhinosinusitis diagnosis rate per 10,000 inhabitants and percentage of acute rhinosinusitis diagnoses with an antibiotic prescription stratified by region and year in Canada, 2007–2013.

Overall a total of 13 million diagnoses of ARS were provided by Canadian physicians between 2007 and 2013. During this time period, the diagnosis rates for ARS decreased from 596 diagnoses per 10,000 inhabitants in 2007 to 464 ARS diagnoses per 10,000 inhabitants in 2013 (Fig 1). Adults (age ≥ 20 years) represented 87.5% of patients diagnosed with ARS in 2013 (Table 1). Furthermore, pediatric patients (age ≤ 19 years) and geriatric patients (age ≥ 60 years) accounted for 11.1% and 15.4% of ARS patients in 2013. During the study period, there was a 44.4% reduction in pediatric ARS cases (1,025 ARS diagnoses per 10,000 inhabitants to 569 ARS diagnoses per 10,000 inhabitants) and a 24.2% reduction in adult ARS cases (2,585 ARS diagnoses per 10,000 inhabitants to 1,960 ARS diagnoses per 10,000 inhabitants) (Table 2). Regional differences were identified, with the highest diagnosis rates for 2013 observed in the Eastern region (557 diagnoses) and the lowest rates in the Western region (366 diagnoses) (Fig 2).

**Table 1:** Diagnoses of acute rhinosinusitis and diagnosis rate per 10,000 inhabitants, antibiotic prescription rate, and percentage of diagnoses with antibiotic prescriptions provided by office-based physicians in Canada by age groups, 2013.

Age groups	Number of diagnoses (%)	Diagnosis rate per 10,000 inhabitants	Antibiotic prescription rate per 10,000 inhabitants	Percentage of diagnoses with antibiotic prescriptions
**0–2**	7,960 (0.5%)	69.9	69.9	100.0%
**3–9**	52,740 (3.2%)	198.3	173.8	87.6%
**10–19**	122,110 (7.4%)	301.6	223.2	74.0%
**Pediatric Total (< 19 yrs)**	**182,810 (11.1%)**	**572.1**	**468.6**	**81.9%**
**20–39**	588,610 (35.8%)	614.3	527.4	85.6%
**40–59**	598,420 (36.4%)	588.7	515.7	87.6%
**60–64**	101,310 (6.2%)	481.2	410.6	85.3%
**65+**	151,040 (9.2%)	281.2	179.2	63.7%
**Geriatric Total (≥ 60 yrs)**	**252,350 (15.4%)**	**762.4**	**589.8**	**77.4%**
**Adult Total (≥ 20 yrs)**	**1,439,380 (87.5%)**	**1965.4**	**1632.9**	**83.1%**
**Age not recorded**	22,710 (1.4%)	N/A	N/A	74.9%
**Total**	**1,644,900 (100%)**	**464.1**	**387.6**	**83.5%**

**Table 2:** Diagnosis rate of acute rhinosinusitis per 10,000 inhabitants and percent change in the diagnosis rate by age group in Canada, 2007 and 2013.

Age groups	Diagnosis rate per 10,000 inhabitants		Percent change in diagnosis rate
	2007	2013	
0–2	363.8	69.5	-80.9%
3–9	346.7	198.3	-42.8%
10–19	314.5	301.6	-4.1%
**Pediatric Total (< 19 years)**	**1025.0**	**569.4**	**-44.4%**
20–39	721.6	612.0	-15.2%
40–59	718.8	586.8	-18.4%
60–64	655.6	480.1	-26.8%
65+	488.5	280.8	-42.5%
**Geriatric Total (≥ 60 yrs)**	**1144.0**	**760.8**	**-33.5%**
**Adult Total (≥ 20 yrs)**	**2584.5**	**1959.7**	**-24.2%**
**Total**	**596.2**	**464.1**	**-22.2%**

During the study period, an overall reduction of 25.0% in antibiotic prescription rates was observed with prescription rates decreasing from 517 antibiotic prescriptions per 10,000 inhabitants to 388 antibiotic prescriptions per 10,000 inhabitants (Table 3). The percentage of diagnoses where an antibiotic was recommended decreased by 3.7% between 2007 and 2013 (from 86.7% to 83.5% of ARS patients) (Fig 1). In 2013, the percentage of ARS patients receiving an antibiotic prescription was highest amongst the 0–2 age group (100% of patients), followed by the 10–19 and 40–59 groupings (both 87.6%) (Table 1). Between 2007 to 2013 the Eastern region had a 15.7% increase in the proportion of patients that were recommended an antibiotic (73.3% to 84.8%) while the the Western region observed a 13.6% decrease (81.6% to 70.5%) (Fig 2). The proportion of patients that received an antibiotic prescription remained unchanged in the Central region (89.7% to 88.1%).

**Table 3:** Percent change in antibiotic prescription rates by Canadian physicians for treating outpatient acute rhinosinusitis by antibiotic class, 2007–2013.

Antibiotic Class	Prescription rate per 10,000 inhabitants		Percent change in antibiotic prescription rate
	2007	2013	
Tetracyclines (Doxycycline and tetracycline)	3.1	8.0	158.1%
Penicillins with extended spectrum (amoxicillin)	115.5	153.5	32.9%
Combinations of penicillins, incl. β-lactamase inhibitors (amoxicillin-clavulanic acid)	31.0	34.9	12.6%
Cephalosporins (overall)	62.1	35.9	-42.2%
First-generation cephalosporins (cefadroxil and cephalexin)	16.6	1.7	-89.8%
Second-generation cephalosporins	39.9	29.8	-25.3%
Cefprozil	18.6	3.8	-79.6%
Cefuroxime	3.3	4.8	45.5%
Cefuroxime axetil	20.8	19.3	-7.2%
Third-generation cephalosporins (cefixime)	1.5	6.9	360.0%
Combinations of sulfonamides and trimethoprim, incl. derivatives (Sulfamethoxazole and sulfadiazine)	12.3	1.7	-86.2%
Macrolides	203.5	105.4	-48.2%
Azithromycin	48.3	29.0	-40.0%
Clarithromycin	147.3	76.4	-48.1%
Fluoroquinolones	87.1	46.8	-46.3%
Ciprofloxacin	8.0	1.6	-80.0%
Levofloxacin	24.9	12.8	-48.6%
Moxifloxacin	54.2	32.4	-40.2%
**All Drugs**	517.0	387.6	-25.0%

**Fig 3:**
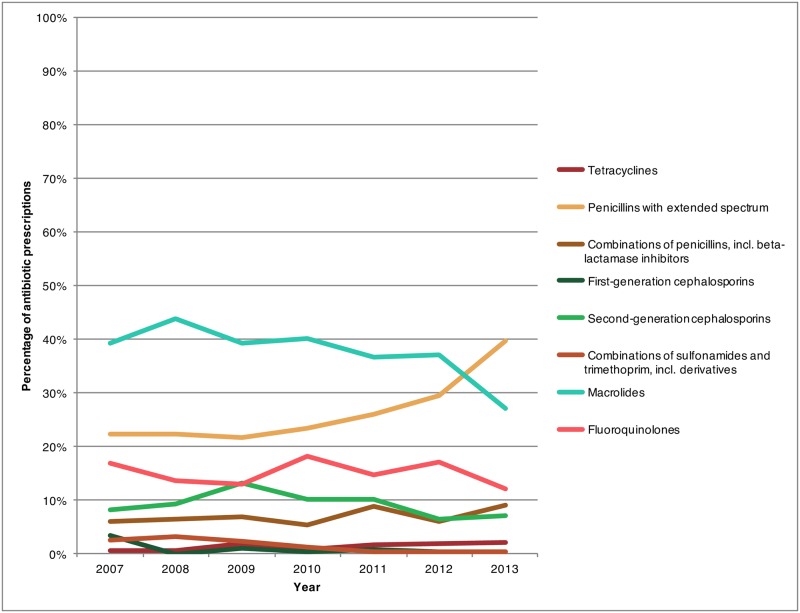
Percentage of antibiotic prescriptions provided by Canadian physicians for treating outpatient acute rhinosinusitis stratified by antibiotic class, 2007–2013.

**Fig 4:**
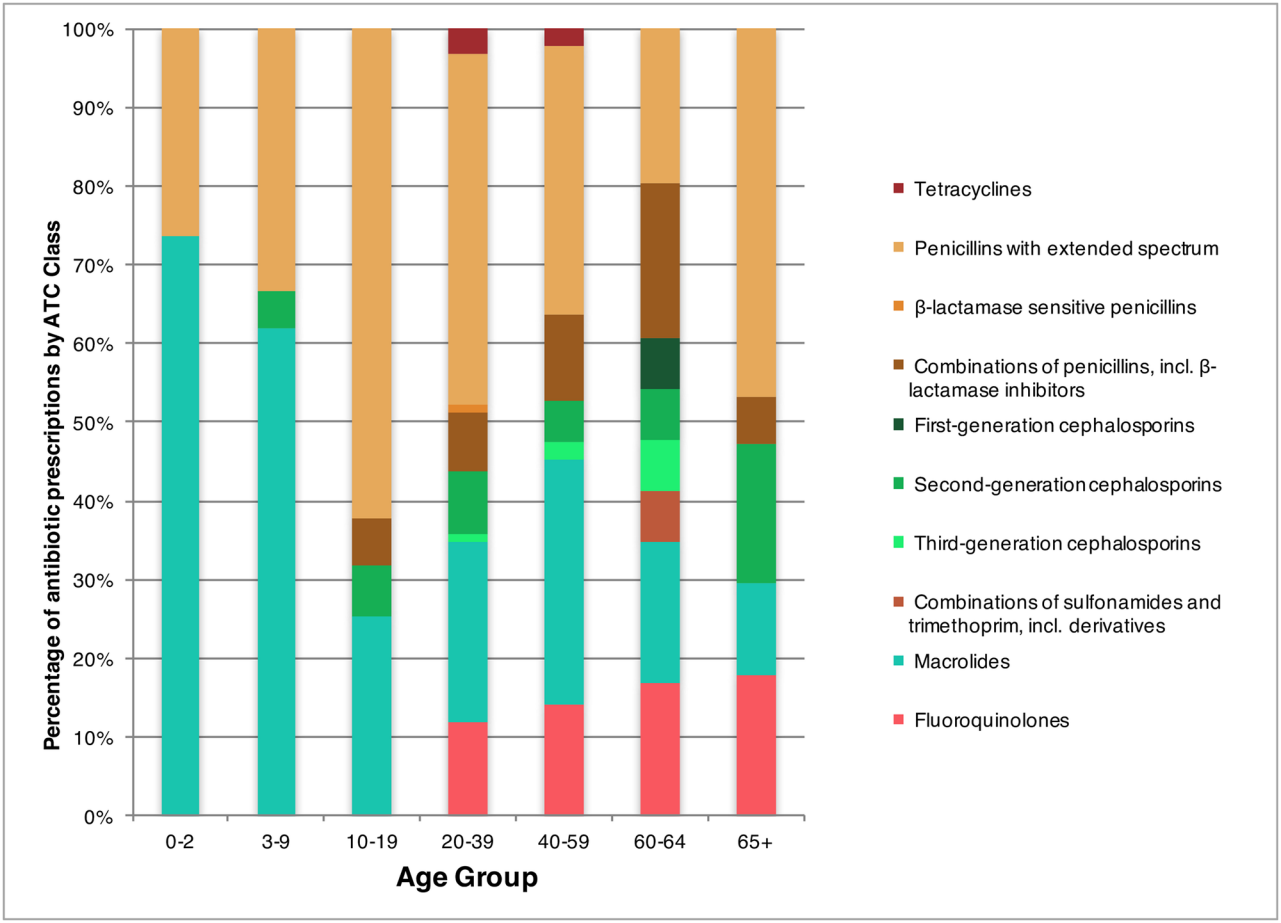
Percentage of antibiotic prescriptions by Canadian physicians for treating outpatient acute rhinosinusitis stratified by antibiotic class and age groups in Canada, 2013. (Footnote: Antibiotic classes were not visualized if they had a mean percentage ≤ 0.6% or ≤ 2 data points. They include the following: β-lactamase resistant penicillins, β-lactamase sensitive penicillins, Third-generation cephalosporins, Lincosamides, and Combinations of sulphonamides and trimethoprim, incl. derivatives).

In 2013, at the national level, the most frequently prescribed antibiotic class for treating ARS switched from macrolides to penicillins with extended spectrum (Fig 3). This switch was not true of all age groups, however, in 2013, macrolides remained the main drug class recommended to the 0–2 year and 3–9 year age groups (73.6% and 62.0% respectively), while for children older than 10 the majority of the prescriptions were for penicillins with extended spectrum (Fig 4). Furthermore, within the 40–59 year age group, there were similar levels of prescription for penicillins with broad spectrum and macrolide class drugs in 2013 (34.0% and 31.1% respectively).

**Fig 5:**
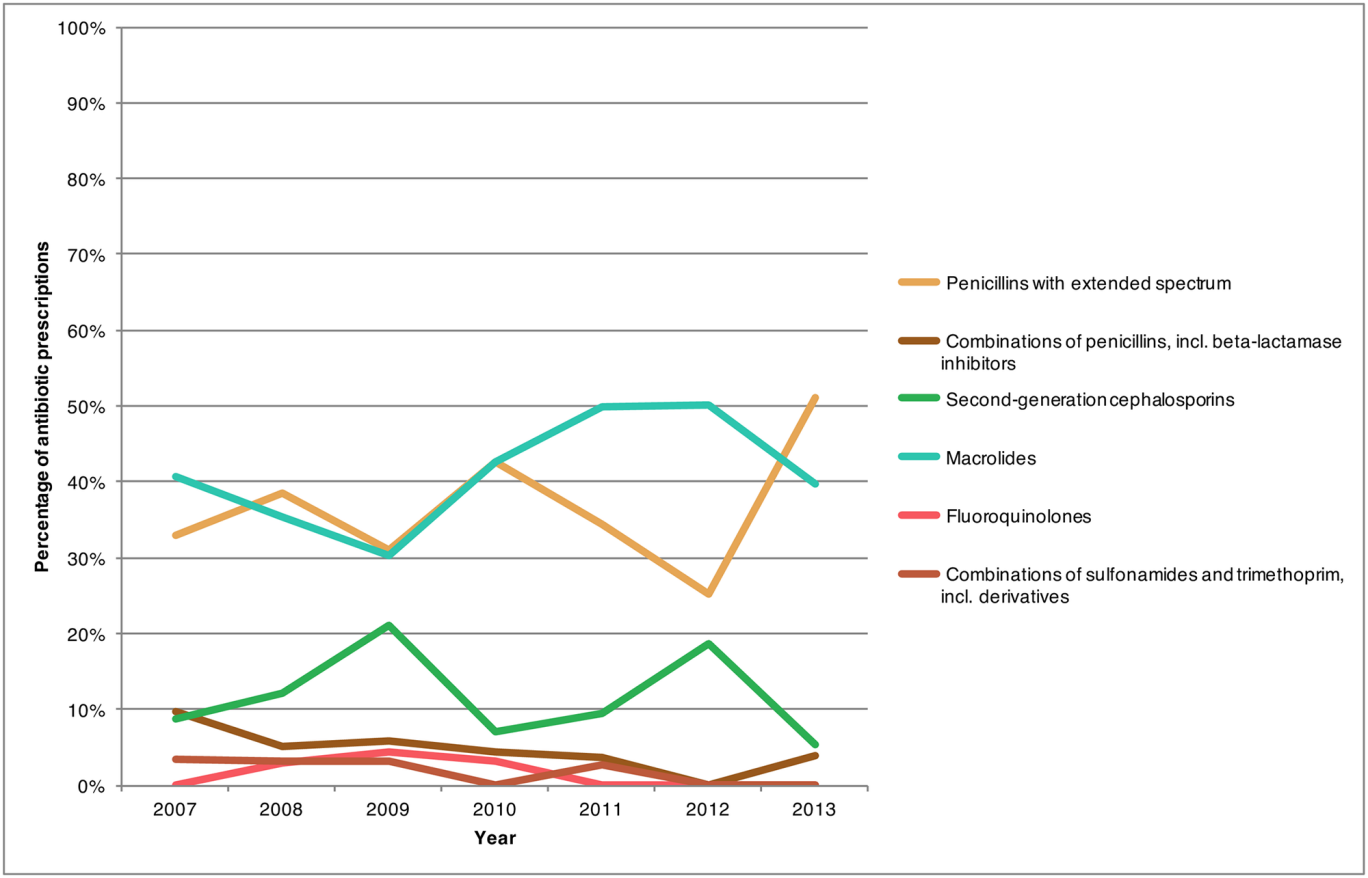
Percentage of antibiotic prescriptions for treating outpatient paediatric (age ≤ 19 yrs.) acute rhinosinusitis by Canadian physicians by antibiotic class, 2007–2013.

The antibiotics prescribed for the treatment of ARS displayed regional variation in 2013 (Table 4). Physicians practicing in the Western and Central regions more commonly recommended amoxicillin for treatment, while physicians in the Eastern region did not have one particular antibiotic of choice. Furthermore, within the Central region, pediatric patients (age ≤ 19 yrs. old) are recommended second-generation cephalosporins (cefprozil and cefuroxime) at a greater rate than combinations of penicillins including a β-lactamase inhibitor (amoxicillin-clavulanic acid) (Fig 5).

**Table 4:** Percent of antibiotic prescriptions for acute rhinosinusitis by antibiotic class stratified by regions in Canada, 2013.

Antibiotics	Region		
	Central	East	West
Tetracyclines	1.8%	N/A	3.9%
Penicillins with extended spectrum (amoxicillin)	40.0%	24.9%	43.6%
β-lactamase sensitive penicillin (phenoxymethylpenicillin)	N/A	N/A	2.0%
Combinations of penicillins, incl. β-lactamase inhibitors (amoxicillin-clavulanic acid)	9.8%	20.2%	2.0%
First gen. cephalosporins (cephalexin)	N/A	4.9%	N/A
Second gen. cephalosporins (cefuroxime and cefprozil)	7.2%	20.0%	2.0%
Third gen. cephalosporins (cefixime)	2.4%	N/A	N/A
Combinations of sulfonamides and trimethoprim, incl. derivatives (sulfamethoxazole and sulfadiazine)	N/A	N/A	2.0%
Macrolides	27.7%	20.2%	28.2%
Fluoroquinolones	11.2%	10.0%	16.0%

## Discussion

To our knowledge this is the first study that describes the levels of ARS diagnosis provided by office-based physicians in Canada and their associated antibiotic prescription practices for treatment of these conditions. The ability to compare the diagnosis rates and antibiotic prescribing rates allowed us to evaluate changes in prescribing practices by physicians while assessing trends of disease over time. An overall decrease in the diagnosis rates were observed during the study period, with the prescribing rates remaining the same over time. However, the impact of new treatment recommendation and guidelines were observed with the change in the antibiotic of preference provided to patients for treatment.

While our results demonstrated a decrease of the ARS diagnosis rate between 2007 and 2013 in Canada, Fairlie *et*. *al* reported that ARS visit rates among American adults between 2000 and 2009 did not change [9]. In our study, adults constituted 88% of patients in 2013, and our inclusion of pediatric ARS cases limits our comparison to the previously mentioned study. While pneumococcal vaccine coverage among Canadian adults is low [6,17], children in Canada have been routinely vaccinated with pneumococcal vaccines since 2006. This routine immunization of children may contribute to the overall herd immunity effect in our country [18,19]. However, pneumococcal vaccines are designed to target specific *Streptococcus pneumonia* serogroups, which could lead to serogroup replacement. The serogroup replacement effect combined with the predominant viral etiology for ARS points to the small role that pneumococcal vaccination programs might have had on the observed ARS diagnosis rate in our study[20]. Nonetheless, the Canadian ARS diagnosis rate declined after 2009, while remaining stable in previous years included in our study (2007–2009). Studies are warranted to further investigate the decrease in the ARS incidence rate observed here and to identify any potential factors which could have contributed to the observed decrease.

The decrease in the number of patients presenting with ARS likely contributed to the simultaneous reduction of antibiotic prescriptions. This decline in antibiotic prescriptions for ARS follows the trend of an overall antibiotic use reduction observed in Canada from 1995 to 2010, while remaining relatively constant between 2010 and 2014s [4,21,22]. While this finding is encouraging, this study found that the percentage of diagnoses with antibiotic prescriptions (indicative of physician prescribing behaviour) did not change, with 84–87% of patients receiving an antibiotic prescription during the study period. These findings are in line with what has been observed in the United States, where more than 80% of acute sinusitis visits have been prescribed an antibiotic for treatment [9,10]. Since 2000, a number of guidelines for treating ARS were produced that recommended increasing the appropriate use of antibiotics [6,7]. However, data on physician prescribing practice indicates that there has not been substantial change following the publication of these guidelines [23].

A considerable variation in antibiotic prescribing practices was observed between the Canadian regions. In the Western Region, both the provinces of British Columbia and Alberta implemented in 2005 a community-based antibiotic stewardship program named “Do Bugs Need Drugs?” [24]. An evaluation of this program was conducted in the metropolitan city of Vancouver which showed that there was a decrease in antibiotic prescribing between 2005 and 2008 [24]. However, the evaluation did not look at the impact in the other Western provinces and it is not possible to determine if similar results were observed. Within the Eastern Region, the province of Newfoundland and Labrador has consistently had the highest antibiotic dispensation rates from community pharmacies in Canada as observed through national surveillance, with the observed prescribing rate in 2014 of 1.0 prescriptions per inhabitant [13]. Overall it is unclear what the contributing factors are to the antibiotic dispensation rates observed in this province, however during the winter of 2016, a community-based antibiotic stewardship campaign targeting patients and physicians was implemented, and it will be interesting to see what impact this has on overall dispensation rates [25].

For most of the study period, macrolide drugs were the most commonly recommended group of antibiotics for ARS patients. However, as of 2011, new national recommendations and guidelines for the treatment of ARS were released [7,26,27] recommending penicillins with broad spectrum as the drug of choice for treatment. The impact of these new recommendations was identified in this study as a change in the drug of preference was observed with macrolide prescriptions decreasing while penicillins with broad spectrum prescriptions increasing in 2013 (Figs 3 and 4). Similarly in the United States penicillins with broad spectrum have become the most common antibiotic class for treating adult and pediatric ARS [9,28]. While drug prescription rates have decreased for most classes of drugs, tetracycline (predominantly doxycycline) prescription rates increased, albeit the absolute increase was small. This may be seen as favourable considering its status as an alternative to amoxicillin as indicated within the Infectious Disease Society of America and other guidelines [6,26,27].

One of the main concerns regarding the inappropriate use of antibiotics is its role in increasing selection pressure for antibiotic resistant pathogens [29]. In light of the high number of diagnoses for ARS that were provided an antibiotic prescription in our study and the fact that the majority of ARS episodes could have a viral etiology, our study has shown that there is room for further improvement in reducing antibiotic prescriptions for uncomplicated ARS cases in Canada [6,7].

Certain limitations present in this data need to be considered interpreting the results. First, there was no clinical information available about the presentations categorized as ARS, leaving room for misclassification of the disease. Furthermore, the ICD-9 label for ARS does not differentiate between ABRS and AVRS; as such we were unable to determine which cases were consistent with ABRS presentations where antibiotics would be appropriate and which were likely AVRS. The high prescribing rate observed might suggest physicians believed cases to be ABRS, however, this data set does not allow for this determination. In addition, information on comorbidities or allergies was not available, reducing our ability to determine if the antibiotic selection was driven by other symptoms or diseases present at the time of diagnosis. Second, the prescribing practices of the small sample of physicians may not be representative of all physicians in Canada. However, the sampling strategy used by IMS Health Canada, Inc. in projecting to national levels has been identified to be fairly accurate when compared with other drug expenditures and health care services.

As the physicians recorded diagnoses and prescriptions in hard copy, there is the potential that errors occurred during transcription to electronic formats. For example, there is the potential that amoxicillin-clavulanic acid was transcribed inaccurately as amoxicillin alone for some records and vice versa, which could have influenced the proportion of prescriptions reported here for both of these products.

## Conclusion

This study found that Canadian physicians commonly recommended antibiotics to ARS patients. This suggests antibiotic treatment of ARS continues to represent a target for improving antimicrobial stewardship and managing antibiotic resistance. Further work should seek to clarify the proportion of ARS patients who have appropriate criteria for antibiotics (ABRS) and which are likely viral presentations (AVRS), where antibiotics could be safely withheld. Such information could help physicians limit unnecessary antibiotic use in ARS treatment and decrease the selection pressure for antibiotic resistance.
